# Prognostic value of FDG PET/CT in special types of breast cancer with non-favorable histology

**DOI:** 10.1007/s12672-025-03278-5

**Published:** 2025-07-31

**Authors:** Mutsumi Fujimoto, Momoko Takaya, Kanako Suzuki, Haruka Ikejiri, Ai Amioka, Emiko Hiraoka, Shinsuke Sasada, Hideo Shigematsu, Morihito Okada

**Affiliations:** Department of Surgical Oncology, Research Institute for Radiation Biology and Medicine, Hiroshima University, 1-2-3-Kasumi, Minami-ku, Hiroshima, 734-8551 Hiroshima Japan

**Keywords:** Special type of breast cancer, FDG PET/CT, SUVmax, Prognostic

## Abstract

**Purpose:**

Prognostic factors for special types of breast cancer remain poorly understood owing to limited clinical research. Therefore, we evaluated the prognostic significance of the maximum standardized uptake value (SUVmax) derived from fluorodeoxyglucose positron emission tomography/computed tomography in patients with special types.

**Methods:**

This retrospective cohort study included patients who underwent curative surgery for special types of breast cancer at Hiroshima University Hospital between 2006 and 2021. Pretreatment SUVmax values, with a cutoff of > 3.0, were evaluated.

**Results:**

Among the 226 identified cases, 105 with favorable histology according to the NCCN guideline were excluded, leaving 121 cases for analysis. The patients had a median follow-up period of 77.8 months (IQR 37.2–114.3). SUVmax > 3.0 was significantly associated with advanced T and N stages, higher nuclear grade, and receipt of adjuvant chemotherapy (*P* = 0.012, *P* = 0.006, *P* < 0.005, *P* < 0.005, respectively). The 5-year relapse-free survival (RFS) and overall survival (OS) rates were 82.8% and 89.2% for SUVmax > 3.0 and 94.6% and 96.7% for SUVmax ≤ 3.0 (*P* = 0.005; *P* = 0.167, respectively). Multivariate analysis identified N stage (Hazard Ratio (HR) 4.20, 95% CI 1.18–14.9, *P* = 0.026) and SUVmax (HR 3.55, 95% CI 1.12–11.2, *P* = 0.026) as significant independent prognostic factors for RFS, while no factors were prognostic for OS.

**Conclusions:**

Our results underscore the utility of SUVmax > 3.0, which is a potential prognostic indicator of poor outcomes in patients with special types of breast cancer, in guiding personalized treatment strategies.

**Supplementary Information:**

The online version contains supplementary material available at 10.1007/s12672-025-03278-5.

## Introduction

Special types of breast cancer, which account for approximately 10% of all invasive breast cancers, are characterized by cytological or histological features distinct from those of invasive ductal carcinoma [1, 2]. The subtypes include invasive lobular, tubular, cribriform and mucinous carcinoma, invasive micropapillary carcinoma, carcinoma with apocrine differentiation, and metaplastic carcinoma. Despite their unique features, randomized controlled trials focusing on these special types of tumors are scarce, resulting in a lack of well-defined prognostic factors and treatment guidelines.

Certain histological types, such as pure mucinous carcinoma, pure tubular carcinoma, and pure cribriform carcinoma, are classified as “favorable histology” by the National Comprehensive Cancer Network (NCCN) Guidelines Version 6.2024 due to their excellent prognosis and low recurrence rates [3]. However, subtypes like metaplastic carcinoma often exhibit aggressive pathological features and poor outcomes, as demonstrated in recent studies [4, 5]. These differences highlight the heterogeneity among special types of breast cancer and underscore the need for further investigation of robust prognostic factors, particularly for non-favorable cancer subtypes.

Fluorodeoxyglucose positron emission tomography/computed tomography (FDG PET/CT) is widely used in breast cancer staging and has demonstrated a prognostic value in invasive breast cancer. Studies have shown that the maximum standardized uptake value (SUVmax) of primary breast tumors is correlated to the tumor growth potential, biological aggressiveness, and lymph node metastasis [6, 7]. Consequently, Kadoya et al. [8] and Xu et al. [9] noted that understanding the prognostic implications of the SUVmax in invasive breast cancer could refine risk stratification and guide treatment decisions. However, its efficacy in specific breast cancer subtypes remains underexplored. Therefore, it is necessary to establish a novel prognostic assessment method to improve the prognostic prediction of special types of breast cancer.

In this study, we aimed to evaluate the prognostic significance of SUVmax in special types of breast cancer, excluding favorable histological types. By focusing on non-favorable histological subtypes, we sought to provide new insights into the utility of FDG PET/CT as a prognostic tool for this under-researched patient population.

## Patients and methods

### Patients

In this retrospective cohort study, we used data from the Hiroshima University Hospital database. We analyzed patients diagnosed with Stage 0–III special type of breast cancer who underwent radical surgery between 2006 and 2021. The inclusion criteria was a pretreatment FDG PET/CT scan with an evaluable breast tumor SUVmax.

In this study, the term “special type of breast carcinoma” was defined as the malignant histological type of “epithelial tumors of the breast,” excluding “invasive breast carcinoma of no special type” and “microinvasive carcinoma.” Furthermore, according to the NCCN guidelines, “favorable histologies” are associated with favorable prognosis, which limits chemotherapy and other systemic therapies. Favorable histology is associated with mucinous, cribriform, tubular, secretory, or adenoid cystic carcinomas. Consequently, these histologic types that were node-negative, human epidermal growth factor receptor 2 (HER2) negative, and not high-grade were excluded from the study [10–14]. In other words, node-positive or HER2 positive or high grade mucinous and tubular carcinomas were included in this study. In addition, according to the NCCN guidelines, encapsulated papillary carcinoma and solid papillary carcinoma are associated with favorable outcomes [15, 16]; therefore, these histologies were excluded from the present study.

This study was approved by the Ethics Review Committee of the Hiroshima University. All procedures performed on human participants were conducted in accordance with the ethical standards of the Institutional Research Committee and the principles of the 1964 Declaration of Helsinki and its later amendments or comparable ethical standards. Given the retrospective nature of this study that used hospital database records, the requirement for written informed consent was waived.

### Clinicopathological assessment

Clinical factors included age at diagnosis, chemotherapy, and radiotherapy. Histological diagnosis was based on the analysis of surgical or biopsy specimens in cases where preoperative chemotherapy had been administered. Tissue classification was based on the fifth edition of the World Health Organization (WHO) classification of tumors. Histological diagnosis, nuclear grade, and lymph node metastasis were determined using hematoxylin and eosin (HE) staining, while estrogen receptor (ER), progesterone receptor (PgR), and HER2 statuses were evaluated by immunostaining in accordance with the American Society of Clinical Oncology (ASCO) guidelines. A sample was considered hormone receptor (HR) positive if > 1% staining was observed in the nucleus, whereas it was considered HER2 positive based on IHC3 + or IHC2 + and FISH + staining. Molecular subtypes were classified as HR + HER2-, HR+/-HER2+, and HR-HER2-. The decision regarding the type of therapy (surgical, pharmacological, or radiation-based) was made at the discretion of the attending physician.

The relapse-free survival (RFS) period was defined as the interval between the date of surgery and the date of diagnosis of recurrence. The overall survival (OS) period was defined as the interval between the date of surgery and the date of confirmed death.

### FDG PET/CT imaging

FDG PET/CT examinations were performed at the same institution using Discovery ST16 PET/CT (GE Healthcare, Little Chalfont, UK).

Patients were required to fast for a minimum of 4 h prior to imaging. Scans were conducted 1–1.5 h after intravenous administration of 3.7 MBq/kg of FDG. Low-dose-weighted CT images (2–4 mm slices) were acquired for attenuation correction and to confirm the location of the lesions identified on the PET images. Immediately after the CT scan, the same axial field of view was scanned with PET for 2–3 min per table position, depending on the patient’s condition and scanner performance. Both PET and CT were performed with the patient in the supine position with normal expiratory breathing. The data were then reconstructed using Fourier rebinning and an ordered subset expectation-maximization algorithm. The region of interest was defined as encompassing the entire intramammary abnormal uptake evident on attenuation-corrected FDG PET images. Quantification of the primary breast tumor and SUVmax was performed using a Xeleris workstation (GE Healthcare).

### Statistical analysis

We used a cutoff of SUVmax > 3.0 to analyze special types of breast cancer, based on our previous study [8]. In the absence of direct references on FDG-PET and prognosis in specific histological subtypes of breast cancer, a T-test was performed on reference 8 and our data in order to evaluate the distribution pattern of SUVmax. We performed a chi-squared statistical test to analyze the distribution of molecular subtypes among histological subtypes. Differences in background factors based on SUVmax values were evaluated using chi-square and Wilcoxon tests. The prognostic significance of SUVmax for RFS and OS was evaluated using the Kaplan–Meier method with a log-rank test. Prognostic factors were evaluated using univariate and multivariate analyses with a Cox proportional hazards model. The Cox proportional hazards model was constructed using the following formula ‘Survival (time, status) ~ SUVmax + N stage + Subtype + Chemotherapy’ to assess the impact of these variables on survival outcomes. Multivariate analyses were evaluated, followed by assessments of time-dependent area under curve (AUC), C-index, interaction effects, variance inflation factor (VIF), and bootstrap validation to ensure model robustness and predictive accuracy. All statistical analyses were performed using JMP Pro 18 software (JMP Statistical Discovery LLC, NC, United States) and the R software (version 4.5.0; https://www.Rproject.org/). Statistical significance was set at *P* < 0.05.

## Results

**Fig. 1:**
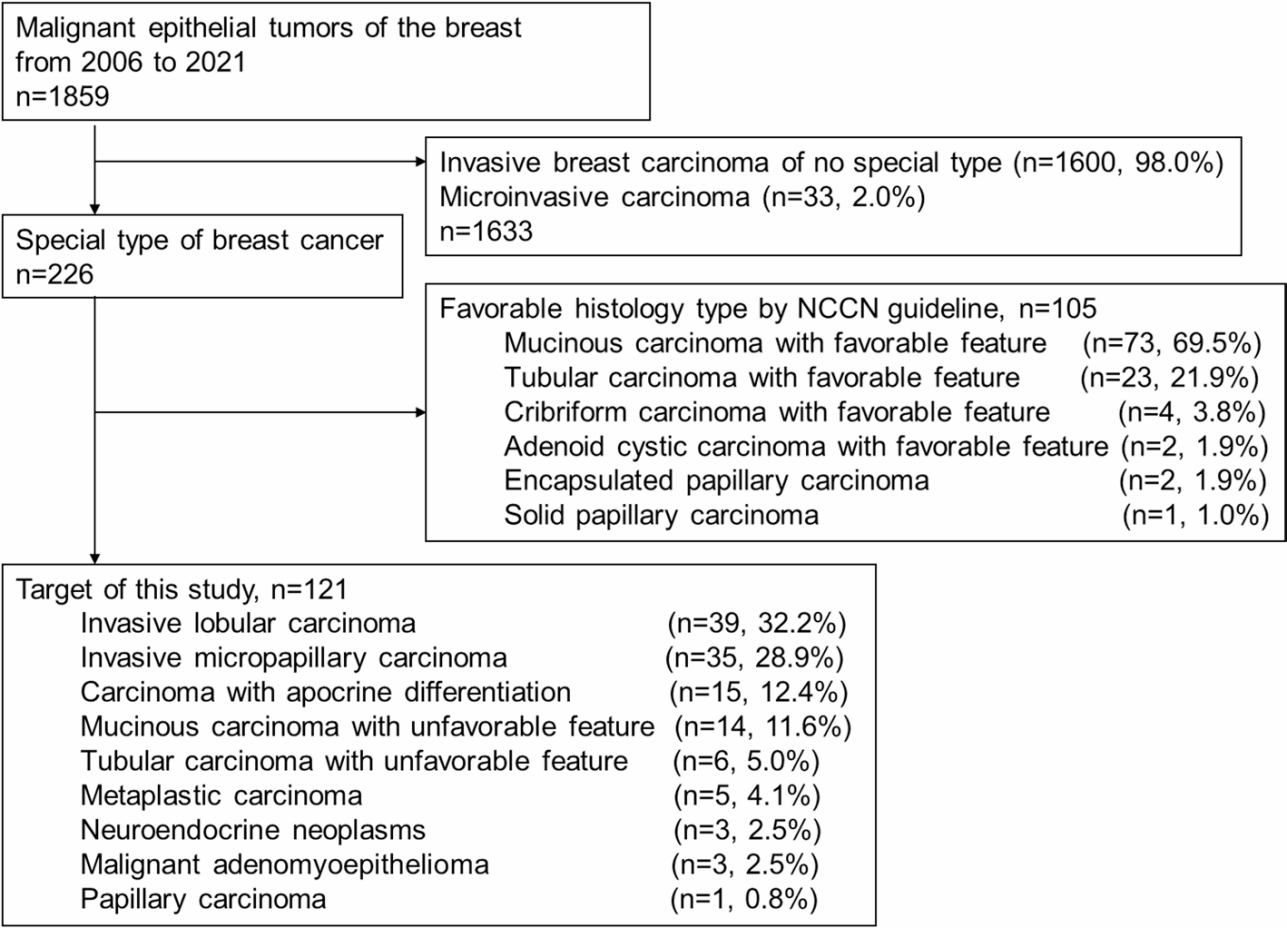
Study flowchart. NCCN: the National Comprehensive Cancer Network

A total of 1859 patients with invasive breast cancer underwent radical surgery between 2006 and 2021. Among them, 1633 (87.8%) patients were identified as having invasive breast carcin“oma of no special type, whereas 226 (12.2%) were classified as having a special type. After excluding 105 (5.6%) patients with “favorable histologies”, the remaining 121 patients (6.6%) with special-type breast cancer were included in the final analysis. (Fig. 1)

The clinicopathological characteristics of the 121 patients are presented in Table 1. Median age was 59.8 years (IQR 49.9–69.1), and median follow up period was 77.8 months (IQR 37.2–114.3). The most prevalent histological type was invasive lobular carcinoma (39 cases, 32.2%), followed by invasive micropapillary carcinoma (35 cases, 28.9%) and carcinoma with apocrine differentiation (15 cases, 12.4%) (Fig. 1). The T and N stages most frequently observed were T1 in 54 (44.6%) patients, and N0 in 68 (56.2%) patients. The observed subtypes were as follows: 91 cases (75.2%) were HR + HER2-, 13 (10.7%) were HR+/-HER2+, and 17 (14.1%) were HR-HER2-. Adjuvant chemotherapy and radiotherapy was administered in 73 (60.3%) and 65 (53.7%) patients, respectively. Table 1 shows the patient backgrounds for each histological type. A subsequent evaluation of previous reports and our own data, employing a T-test, demonstrated a comparable distribution pattern for SUVmax (Table S1). Notably, the prevalence of HER2-positive mucinous carcinoma was higher, because luminal-type and low-grade mucinous carcinomas were excluded owing to their favorable prognosis. Additionally, triple-negative carcinomas were more prevalent among carcinomas with apocrine differentiation, adenomyoepithelial carcinomas and papillary carcinomas compared to other histologies (Table S2).

**Table 1:** Characteristics of all patients and each histological type

		All		Lobular		Micropapillary		Apocrine		Mucinous		Tubular		Metaplastic		Neuroendocrine		Adenomyoepithelial		Papillary	
		*n* = 121		*n* = 39		*n* = 35		*n* = 15		*n* = 14		*n* = 6		*n* = 5		*n* = 3		*n* = 3		*n* = 1	
Age (Median, IQR)		59.8 (49.9–69.1)		61.8 (51.0-67.8)		56.7 (45.1–69.5)		68.7 (59.9–74.3)		56.3 (48.7–75.6)		53.1 (47.2–57.1)		64.0 (52.3–71.3)		49.3 (46.9–57.9)		55.9 (33.0-72.4)		62.3	
T Stage	T1	54	(44.6%)	13	(33.3%)	17	(48.6%)	11	(73.3%)	5	(35.7%)	3	(50.0%)	2	(40.0%)	1	(33.3%)	1	(33.3%)	1	(100%)
	T2	48	(39.7%)	15	(38.5%)	12	(34.3%)	4	(26.7%)	8	(57.1%)	3	(50.0%)	2	(40.0%)	2	(66.7%)	2	(66.7%)	0	
	T3	11	(9.1%)	7	(18.0%)	3	(8.6%)	0		1	(7.1%)	0		0		0		0		0	
	T4	8	(6.6%)	4	(10.2%)	3	(8.6%)	0		0		0		1	(20.0%)	0		0		0	
N Stage	N0	68	(56.2%)	22	(56.4%)	13	(37.1%)	15	(100%)	9	(64.3%)	3	(50.0%)	2	(40.0%)	1	(33.3%)	2	(66.7%)	1	(100%)
	N1	33	(27.3%)	9	(23.1%)	14	(40.0%)	0		3	(21.4%)	3	(50.0%)	2	(40.0%)	1	(33.3%)	1	(33.3%)	0	
	N2	14	(11.5%)	8	(20.5%)	4	(11.4%)	0		2	(14.3%)	0		0		0		0		0	
	N3	6	(5.0%)	0		4	(11.4%)	0		0		0		1	(20.0%)	1	(33.3%)	0		0	
Nuclear grade	NG1	27	(22.3%)	18	(46.2%)	5	(14.3%)	3	(20.0%)	0		0		0		0		1	(33.3%)	0	
	NG2	41	(33.9%)	16	(41.0%)	14	(40.0%)	5	(33.3%)	4	(28.6%)	0		1	(20.0%)	0		1	(33.3%)	0	
	NG3	52	(43.0%)	5	(12.8%)	16	(45.7%)	7	(46.7%)	10	(71.4%)	6	(100%)	4	(80.0%)	3	(100%)	0		1	(100%)
	Unknown	1	(0.8%)	0		0		0		0		0		0		0		1	(33.3%)	0	
ER	Positive	99	(81.8%)	37	(24.9%)	34	(97.1%)	5	(33.3%)	12	(85.7%)	6	(100%)	2	(40.0%)	2	(66.7%)	1	(33.3%)	0	
	Negative	22	(18.2%)	2	(5.1%)	1	(2.9%)	10	(66.7%)	2	(14.3%)	0		3	(60.0%)	1	(33.3%)	2	(66.7%)	1	(100%)
PgR	Positive	91	(75.2%)	33	(84.6%)	33	(94,3%)	3	(20.0%)	11	(78.6%)	6	(100%)	2	(40.0%)	2	(66.7%)	1	(33.3%)	0	
	Negative	30	(24.8%)	6	(15.4%)	2	(5.7%)	12	(80.0%)	3	(21.4%)	0		3	(60.0%)	1	(33.3%)	2	(66.7%)	1	(100%)
HER2	Positive	13	(10.7%)	1	(2.6%)	4	(11.4%)	2	(13.3%)	9	(64.3%)	1	(16.7%)	0		0		0		0	
	Negative	108	(89.3%)	38	(97.4%)	31	(88.6%)	13	(86.7%)	5	(35.7%)	5	(83.3%)	5	(100%)	3	(100%)	3	(100%)	1	(100%)
Subtype	HR + HER-	91	(75.2%)	36	(92.3%)	31	(88.6%)	3	(20.0%)	9	(64.3%)	5	(83.3%)	3	(60.0%)	3	(100%)	1	(33.3%)	0	
	HER+	13	(10.7%)	1	(2.6%)	4	(11.4%)	2	(13.3%)	5	(35.7%)	1	(16.7%)	0		0		0		0	
	HR-HER-	17	(14.1%)	2	(5.1%)	0		10	(66.7%)	0		0		2	(40.0%)	0		2	(66.7%)	1	(100%)
Chemotherapy	Yes	73	(60.3%)	20	(51.3%)	23	(65.7%)	6	(40.0%)	7	(50.0%)	6	(100%)	5	(100%)	3	(100%)	3	(100%)	0	
	No	48	(39.7%)	19	(48.7%)	12	(24.3%)	9	(60.0%)	7	(50.0%)	0		0		0		0		1	(100%)
Radiotherapy	Yes	65	(53.7%)	22	(56.4%)	24	(68.6%)	5	(33.3%)	7	(50.0%)	3	(50.0%)	1	(20.0%)	2	(66.7%)	1	(33.3%)	0	
	No	56	(46.3%)	17	(43.6%)	11	(31.4%)	10	(66.7%)	7	(50.0%)	3	(50.0%)	4	(80.0%)	1	(33.3%)	2	(66.7%)	1	(100%)

*IQR* interquartile range, *NG* nuclear grade, *ER* estrogen receptor, *PgR* progesterone receptor, *HR* hormone receptor, *HER2* human epidermal growth factor receptor 2 SUVmax Maximum standardized uptake value.

**Fig. 2:**
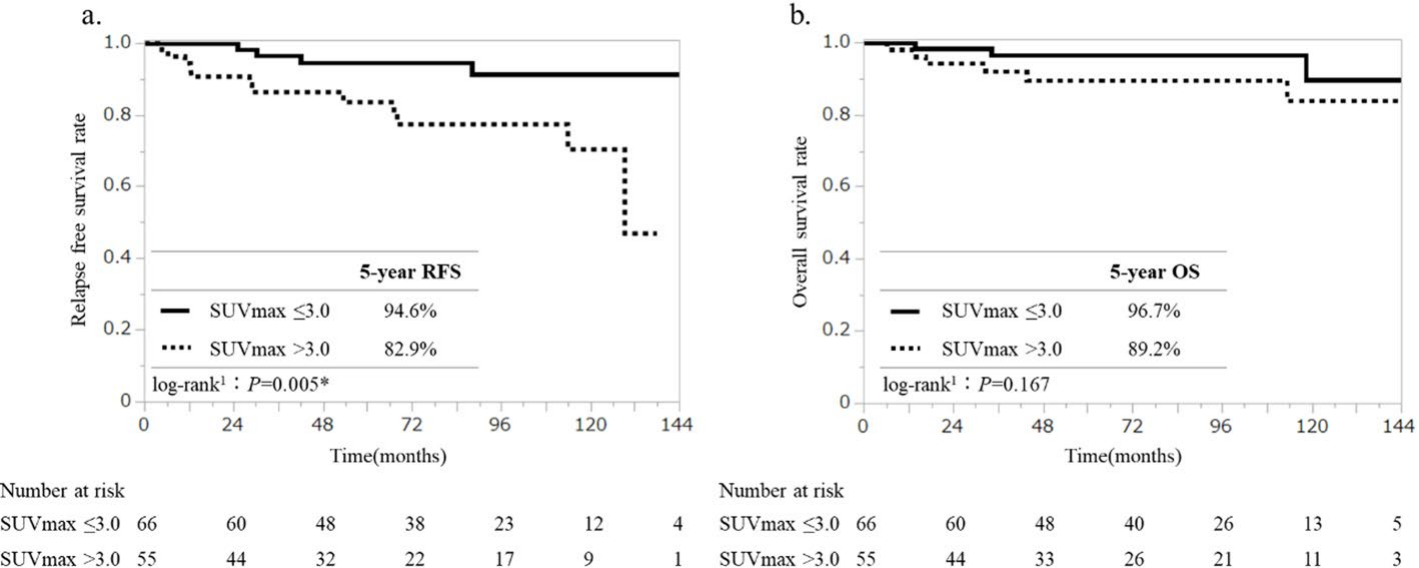
Relapse free survival curve and Overall survival of patients according to SUVmax values. **a** Relapse free survival and **b** Overall survival of all patients. RFS relapse free survival, OS overall survival, SUVmax maximum standardized uptake value. ^1^ Log-rank test, *significant *P*-value < 0.05

Overall, 66 patients (54.5%) had SUVmax ≤ 3.0 and 55 patients (45.5%) had SUVmax > 3.0. Additionally, SUVmax > 3.0 was significantly associated with higher T stage, N stage, higher nuclear grade, and adjuvant chemotherapy than SUVmax ≤ 3.0 in Fisher’s exact test (*P* = 0.012, *P* = 0.006, *P* < 0.005, *P* < 0.005, respectively). (Table 2) Among all patients, recurrence occurred in 16 (13.2%) while nine (7.4%) died. The 5-year RFS was 94.6% (SE ± 3.02%) for SUVmax ≤ 3.0 and 82.9% (SE ± 5.63%) SUVmax > 3.0 (*P* = 0.005). The 5-year OS was 96.7% (SE ± 2.32%) for SUVmax ≤ 3.0 and 89.2% (SE ± 4.61%) SUVmax > 3.0 (*P* = 0.167) (Fig. 2a, b). The prognosis for RFS was statistically significantly more favorable when the SUVmax was ≤ 3.0.

**Table 2:** Comparison of clinicopathological parameters between suvmax ≤ 3.0 and > 3.0

		SUVmax ≤ 3.0 (*n* = 66)		SUVmax > 3.0 (*n* = 55)		*P* ^1^
age (Median, IQR)		60.8 (51.1–68.7)		57.9 (46.6–69.5)		0.340^2^
T Stage	T1	38	(57.6%)	16	(29.1%)	0.012*
	T2	19	(28.8%)	29	(52.7%)	
	T3	6	(9.0%)	5	(9.1%)	
	T4	3	(4.5%)	5	(9.1%)	
N Stage	N0	43	(65.2%)	25	(45.5%)	0.006*
	N1	17	(25.8%)	16	(29.1%)	
	N2	6	(9.0%)	8	(14.5%)	
	N3	0		6	(10.9%)	
Nuclear grade	NG1	23	(34.9%)	4	(7.3%)	< 0.005*
	NG2	22	(33.3%)	19	(34.5%)	
	NG3	20	(30.3%)	32	(58.2%)	
	Unknown	1	(1.5%)	0		
ER	Positive	53	(80.3%)	46	(83.6%)	0.635
	Negative	13	(19.7%)	9	(16.4%)	
PgR	Positive	50	(75.8%)	41	(74.6%)	0.878
	Negative	16	(24.2%)	14	(25.4%)	
HER2	Positive	5	(7.6%)	8	(14.5%)	0.218
	Negative	61	(92.4%)	47	(85.5%)	
Subtype	HR + HER-	52	(78.8%)	39	(71.0%)	0.445
	HR + HER+	5	(7.6%)	8	(14.5%)	
	HR-HER-	9	(13.6%)	8	(14.5%)	
Chemotherapy	Yes	32	(48.5%)	41	(74.5%)	< 0.005*
	No	34	(51.5%)	14	(25.5%)	
Radiotherapy	Yes	35	(53.0%)	30	(54.6%)	0.868
	No	31	(47.0%)	25	(45.4%)	

*SUVmax* maximum standardized uptake value, *IQR* interquartile range, *NG* nuclear grade, *ER* estrogen receptor, *PgR* progesterone receptor, *HR* hormone receptor, *HER2* human epidermal growth factor receptor 2.

^1^ Chi-square *P*-value, ^2^ Wilcoxon rank-sum test, * significant *P*-value < 0.05.

Table 3 presents the 5-year RFS and OS rates according to the SUVmax for each histological type. Over the five-year period, no cases of recurrence or mortality due to invasive lobular carcinoma, mucinous carcinoma, tubular carcinoma, encapsulated papillary carcinoma, solid papillary carcinoma, or papillary carcinoma were observed. However, three cases of recurrence were observed in patients with invasive lobular carcinoma after a ten-year period. In the context of micropapillary carcinomas, the 5-year RFS rate was 93.8% for SUVmax ≤ 3.0 and 80.1% for SUVmax > 3.0, and the 5-year OS was 94.1% for SUVmax ≤ 3.0 and 93.3% SUVmax > 3.0. In the case of apocrine carcinomas, the 5-year RFS was 100% for SUVmax ≤ 3.0 and 80.0% for SUVmax > 3.0. No cases of mortality were observed (Table 3). Subsequent evaluation of prognostic differences based on the SUVmax was conducted for each molecular subtype (Table 4). In the HR + HER2-subtype, the 5-year RFS was 95.2% (SE ± 3.29%) for SUVmax ≤ 3.0 and 87.4% (SE ± 6.07%) for SUVmax > 3.0 (*P* = 0.020), indicating a statistically significantly better prognosis with SUVmax ≤ 3.0. Whereas no statistically significant differences were observed between the HR+/- HER2 + and HR-HER2- subtypes. Furthermore, no substantial variations in 5-year OS were identified among the subtypes.

**Table 3:** Five-year RFS and OS according to SUVmax for each histological type

	All	Lobular	Micropapillary	Apocrine	Mucinous	Tubular	Metaplastic	Neuroendocrine	Adenomyoepithelial	Papillary
	(*n* = 121)	(*n* = 39)	(*n* = 35)	(*n* = 15)	(*n* = 14)	(*n* = 6)	(*n* = 5)	(*n* = 3)	(*n* = 3)	(*n* = 1)
5-year RFS										
SUVmax ≤ 3.0	94.6%	100%	93.8%	100%	100%	100%	-	0%	0%	100%
SUVmax > 3.0	82.8%	100%	80.1%	80.0%	100%	100%	40%	50.0%	100%	-
5-year OS										
SUVmax ≤ 3.0	96.7%	100%	94.1%	100%	100%	100%	-	0%	100%	100%
SUVmax > 3.0	89.2%	100%	93.3%	100%	100%	100%	40%	50.0%	100%	-

*RFS* relapse free survival, *OS* overall survival, *SUVmax* maximum standardized uptake value.

**Table 4:** Five-year RFS and OS according to SUVmax for each molecular subtype

	HR + HER2-	HR+/-HER2+	HR-HER2-
	(*n* = 91)	(*n* = 13)	(*n* = 17)
5-year RFS			
SUVmax ≤ 3.0	95.2%	100%	87.5%
SUVmax > 3.0	87.4%	87.5%	57.1%
5-year OS			
SUVmax ≤ 3.0	95.7%	100%	100%
SUVmax > 3.0	90.6%	100%	71.4%

*RFS* relapse free survival, *OS* overall survival, *SUVmax* maximum standardized uptake value, *HR* hormone receptor, *HER2* human epidermal growth factor receptor 2.

The univariate analysis identified nodal metastasis (HR 5.19, 95% CI 1.48–18.2, *P* = 0.010) and SUVmax (HR 1.11, 95% CI 1.05–1.18, *P* < 0.005) as factors potentially associated with RFS (Table 5). Subsequently, in the multivariate analysis model included clinically important subtypes and the presence or absence of chemotherapy, in addition to SUVmax and N stage, which showed statistical significance in the univariate analysis. SUVmax (HR 1.13, 95% CI 1.05–1.23, *P* < 0.005), nodal metastasis (HR 2.62, 95% CI 1.57–4.35, *P* < 0.005) and HR-HER- (HR 1.13, 95% CI 1.87–1.23, *P* < 0.005) were significantly associated with RFS (Table 5). In the multivariable Cox regression analysis, the interaction term between SUVmax and nodal status was not statistically significant (HR = 0.85, 95% CI: 0.05–13.3, *P* = 0.909), indicating that the effect of SUVmax does not significantly differ depending on the presence of nodal status. In addition, time-dependent AUCs at 1, 3, and 5 years were 0.74, 0.69, and 0.71, respectively. The multivariate Cox model including SUVmax and nodal status, Harrell’s concordance index (C-index) was calculated. The C-index for relapse-free survival prediction was 0.728, indicating acceptable discriminative ability. The VIF values for N factor, Subtype, SUVmax, and CT factor were 1.28, 1.36, 1.06, and 1.22, respectively, all of which are well below the conventional threshold of 5. Internal validation using 1000 bootstrap resamples was performed to assess the stability of the Cox proportional hazards model. The results indicated a 95% confidence interval of 0.171–1.443. In the context of overall survival, the univariate analysis identified SUVmax (HR 1.14, 95% CI 1.05–1.23 *P* < 0.005) as factors potentially associated with OS. In the multivariate analysis model used the same factors of RFS analysis. SUVmax (HR 1.11, 95% CI 1.00–1.24, *P* = 0.040) demonstrated a significant association with OS (Table 6).

**Table 5:** Univariate analysis and multivariate analysis of association of relapse free survival

		Univariate analysis		Multivariate analysis	
		Hazard ratio (95%CI)	*P* value^1^	Hazard ratio (95%CI)	*P* value^1^
age	> 60	Reference			
	≤ 60	2.57 (0.82–7.97)	0.103		
T Stage	T1	Reference			
	T2, T3, T4	2.31 (0.74–7.17)	0.148		
N Stage	N0	Reference		Reference	
	N1, N2, N3	5.19 (1.48–18.2)	0.010*	2.62 (1.57–4.35)	< 0.005*
Nuclear grade	NG1, NG2	Reference			
	NG3	1.60 (0.58–4.41)	0.367		
Subtype	HR + HER-	Reference		Reference	
	HER+	0.57 (0.07–4.45)	0.596	0.52 (0.07–4.12)	0.546
	HR-HER-	2.09 (0.67–6.59)	0.209	8.00 (1.87–34.1)	< 0.005*
SUVmax	≦ 3.0	Reference			
	> 3.0	4.40 (1.42–13.7)	0.010*		
	continuous	1.11 (1.05–1.18)	< 0.005*	1.13 (1.05–1.23)	< 0.005*
Chemotherapy	No	Reference		Reference	
	Yes	4.21 (0.96–18.6)	0.057	1.92 (0.38–9.66)	0.431
Radiotherapy	No	Reference			
	Yes	1.47 (0.55–3.95)	0.440		

*CI* confidential interval, *NG* nuclear grade, *HR* hormone receptor, *HER2* human epidermal growth factor receptor 2, *SUVmax* maximum standardized uptake value, *NA* not available.

^1^ Cox proportional hazards model, *significant *P*-value < 0.05.

**Table 6:** Univariate analysis and multivariate analysis of association of overall survival

		Univariate analysis		Multivariate analysis	
		Hazard ratio (95%CI)	*P* value^1^	Hazard ratio (95%CI)	*P* value^1^
age	> 60	Reference			
	≤ 60	2.87 (0.59–13.9)	0.190		
T Stage	T1	Reference			
	T2, T3, T4	2.77 (0.57–13.4)	0.204		
N Stage	N0	Reference		Reference	
	N1, N2, N3	3.89 (0.80–18.8)	0.091	1.76 (0.95–3.26)	0.072
Nuclear grade	NG1, NG2	Reference			
	NG3	1.74 (0.46–6.48)	0.412		
Subtype	HR + HER-	Reference		Reference	
	HER+	NA		NA	
	HR-HER-	1.68 (0.35–8.12)	0.516	2.16 (0.39-12.0)	0.380
SUVmax	≦ 3.0	Reference			
	> 3.0	2.57 (0.64–10.3)	0.182		
	continuous	1.14 (1.05–1.23)	< 0.005*	1.11 (1.00-1.24)	0.040*
Chemotherapy	No	Reference		Reference	
	Yes	4.44 (0.55–35.7)	0.160	2.42 (0.26–22.9)	0.441
Radiotherapy	No	Reference			
	Yes	1.79 (0.48–6.71)	0.388		

*CI* confidential interval, *NG* nuclear grade, *HR* hormone receptor, *HER2* human epidermal growth factor receptor 2, *SUVmax* maximum standardized uptake value, *NA* not available.

^1^ Cox proportional hazards model, *significant *P*-value < 0.05.

## Discussion

To the best of our knowledge, this study represents the first investigation of the potential of PET/CT as a prognostic factor for special types of breast cancer. Factors associated with recurrence were identified using univariate and multivariate analysis. Patients in the low-risk group with SUVmax ≤ 3.0 had a more favorable prognosis compared to those with SUVmax > 3.0. Therefore, these patients may benefit from a reduction in postoperative therapy to minimize the adverse effects. Conversely, the presence of positive lymph nodes and an SUVmax > 3.0 were identified as significant prognostic factors. Additionally, these patients had an elevated risk of recurrence, and thus should be considered a high-risk patient group. SUVmax has the potential to function as a risk stratification biomarker.

Each histological type of breast cancer is characterized by distinct clinicopathologic features [17]. Some histological types are associated with favorable prognosis and low recurrence rates [18]. The NCCN guidelines categorize pure mucinous, ductal, and cribriform carcinoma, which are HER2 negative and not high grade, as histologic types with a good prognosis [3, 19]. Therefore, these histological types were excluded from this study. At our institution, 105 patients with special types of breast cancer with favorable prognoses were excluded from the study. Moreover, only one (1%) patient developed recurrence during the same follow-up period as in the present study, indicating a favorable prognosis. The exclusion of favorable histologies facilitated a more comprehensive investigation into the heterogeneity and prognostic value of SUVmax in a cohort of cases that is more clinically relevant.

The analysis demonstrates relatively high OS even within the group exhibiting high SUVmax. This outcome may be indicative of the efficacy of the treatment options available at our institution. It is important to consider other potential causative factors, including the possibility of bias resulting from histologic type. The study incorporated a total of five cases of chemogenic carcinoma, a histologic type that is recognized for its unfavorable prognosis.

Similar to prognosis, the degree of FDG PET/CT accumulation also varies by histology [20]. FDG accumulation is related to cell density. Pure mucinous carcinoma has a low FDG uptake possibly due to high extracellular mucin content and low cell density, which may explain its favorable prognosis [21]. Additionally, invasive lobular carcinoma typically exhibit lower SUVmax values than IDCs [22]. This phenomenon is attributed to the diffuse infiltrative growth pattern of invasive lobular carcinoma, low tumor cell density, low glucose transporter-1 (GLUT1) expression levels, and low growth rate [23]. Furthermore, this phenomenon correlates with the tumor diameter and nuclear grade, indicating its possible prognostic significance. Conversely, metaplastic carcinomas are characterized by several aggressive pathological features and are associated with a poor prognosis [4, 5]. Likewise, a previous study reported that most cases of metaplastic carcinomas were triple-negative, and had relatively high PET/CT accumulation [24].

PET/CT is a diagnostic test that targets glucose metabolism. In breast cancer, FDG uptake is correlated with GLUT1 protein expression [25, 26]. Thus, in some types of breast cancer, the expression of molecules related to glucose metabolism, such as GLUT1, may be indicative of differences in PET/CT accumulation, owing to differences in tumor metabolism. Moreover, microvessel density, lymphocyte count, and necrosis may be associated with the microenvironment. This may facilitate the development of therapies targeting these factors in high-risk groups. However, exploring the differences in SUVmax and the molecular basis of the individual histological types of other types of breast cancer is a future challenge.

Despite its positive outcomes, the study has some limitations. First, this was a single-center retrospective analysis, which inherently limits the generalizability of the findings. Additionally, the retrospective nature of the study introduced potential selection bias and confounding factors that could not be fully adjusted for. Second, SUVmax values and cut-off points may vary between institutions owing to differences in imaging protocols and equipment. Therefore, multicenter studies with standardized PET/CT protocols and prospective designs are necessary to validate the prognostic utility of the SUVmax for special types of breast cancer. Despite these limitations, this study provides an important first step toward understanding the prognostic value of the SUVmax in rare and underrepresented patient groups.

## Conclusion

Higher SUVmax was significantly associated with breast cancer recurrence in patients with specific types of breast cancer, excluding those with a favorable prognosis. This suggests that the SUVmax may be a useful prognostic indicator in this patient group. The results of this study are expected to improve the accuracy of prognostic prediction and individualize treatment strategies.

## Supplementary Information

Below is the link to the electronic supplementary material.

Supplementary Material 1

## Data Availability

All supplementary data generated or analyzed during this study are included in the supplementary file published alongside this manuscript.

## References

[1] Cserni G. Histological type and typing of breast carcinomas and the WHO classification changes over time. Pathologica. 2020;112:25–41. 10.32074/1591-951X-1-20. 32202537 10.32074/1591-951X-1-20 PMC8138497

[2] Kim J, Kim JY, Lee HB, Lee YJ, Seong MK, Paik N, et al. Characteristics and prognosis of 17 special histologic subtypes of invasive breast cancers according to world health organization classification: comparative analysis to invasive carcinoma of no special type. Breast Cancer Res Treat. 2020;184:527–42. 10.1007/s10549-020-05861-6. 32794061 10.1007/s10549-020-05861-6

[3] National Comprehensive Cancer Network. Breast cancer. 2024. https://www.nccn.org/professionals/physician_gls/pdf/breast.pdf Accessed 3 Dec 2024 (version 6; 2024).

[4] Miao Z, Ba F, Wen Z, Chen K, Shen X, Gen F, et al. Survival trends of patients with metaplastic breast carcinoma with different hormone receptor statuses: a SEER-based retrospective cohort study. BMC Womens Health. 2024;24:628. 10.1186/s12905-024-03470-9. 39593066 10.1186/s12905-024-03470-9 PMC11590209

[5] Du J, Wu S, Liu J, Guo B, Li J, Li W, et al. Analysis of clinicopathological characteristics and prognostic factors in 54 metaplastic breast carcinoma patients from Northwest China. CytoJournal. 2024;21:31. 10.25259/Cytojournal_15_2024. 39411170 10.25259/Cytojournal_15_2024 PMC11474753

[6] Sollini M, Cozzi L, Ninatti G, Antunovic L, Cavinato L, Chiti A, et al. PET/CT radiomics in breast cancer: Mind the step. Methods. 2021;188:122–32. 10.1016/j.ymeth.2020.01.007. 31978538 10.1016/j.ymeth.2020.01.007

[7] Cheng J, Ren C, Liu G, Shui R, Zhang Y, Li J, et al. Development of high-resolution dedicated PET-based radiomics machine learning model to predict axillary lymph node status in early-stage breast cancer. Cancers. 2022;14:950. 10.3390/cancers14040950. 35205699 10.3390/cancers14040950 PMC8870230

[8] Kadoya T, Aogi K, Kiyoto S, Masumoto N, Sugawara Y, Okada M. Role of maximum standardized uptake value in Fluorodeoxyglucose positron emission tomography/computed tomography predicts malignancy grade and prognosis of operable breast cancer: A multi-institute study. Breast Cancer Res Treat. 2013;141:269–75. 10.1007/s10549-013-2687-7. 24026860 10.1007/s10549-013-2687-7 PMC3785187

[9] Xu X, Sun X, Ma L, Zhang H, Ji W, Xia X, et al. 18F-FDG PET/CT radiomics signature and clinical parameters predict progression-free survival in breast cancer patients: a preliminary study. Front Oncol. 2023;13:1149791. 10.3389/fonc.2023.1149791. 36969043 10.3389/fonc.2023.1149791 PMC10036789

[10] Zhou X, Zheng Z, Li Y, Zhao W, Lin Y, Zhang J, et al. The clinical features and prognosis of patients with mucinous breast carcinoma compared with those with infiltrating ductal carcinoma: a population-based study. BMC Cancer. 2021;21:536. 10.1186/s12885-021-08262-0. 33975551 10.1186/s12885-021-08262-0 PMC8111957

[11] Liu J, Zheng X, Han Z, Lin S, Han H, Xu C. Clinical characteristics and overall survival prognostic nomogram for invasive cribriform carcinoma of breast: a SEER population-based analysis. BMC Cancer. 2021;21:168. 10.1186/s12885-021-07895-5. 33593316 10.1186/s12885-021-07895-5 PMC7887783

[12] Metovic J, Bragoni A, Osella-Abate S, Borella F, Benedetto C, Gualano MR, et al. Clinical relevance of tubular breast carcinoma: large retrospective study and meta-analysis. Front Oncol. 2021;11:653388. 10.3389/fonc.2021.653388. 33996576 10.3389/fonc.2021.653388 PMC8117349

[13] Gong P, Xia C, Yang Y, Lei W, Yang W, Yu J, et al. Clinicopathologic profiling and oncologic outcomes of secretory carcinoma of the breast. Sci Rep. 2021;11:14738. 10.1038/s41598-021-94351-w. 34282256 10.1038/s41598-021-94351-w PMC8289843

[14] Neilson T, Li Z, Minami C, Myers SP. Adenoid cystic carcinoma of the breast: a narrative review and update on management. Cancers. 2025;17(7):1079. 10.3390/cancers17071079. 40227583 10.3390/cancers17071079 PMC11988025

[15] Rakha EA, Gandhi N, Climent F, van Deurzen CH, Haider SA, Dunk L, ey al. Encapsulated papillary carcinoma of the breast: an invasive tumor with excellent prognosis. Am J Surg Pathol. 2011;35(8):p1093–1103. 10.1097/PAS.0b013e31821b3f65. 10.1097/PAS.0b013e31821b3f65 21753694

[16] Jinous S, Marilin R. Solid papillary carcinoma of the breast: a pathologically and clinically distinct breast tumor. Arch Pathol Lab Med. 2012;136(10):1308–11. 10.5858/arpa.2011-0227-RS. 23020734 10.5858/arpa.2011-0227-RS

[17] Li CI, Uribe DJ, Daling JR. Clinical characteristics of different histologic types of breast cancer. Br J Cancer. 2005;93:1046–52. 10.1038/sj.bjc.6602787. 16175185 10.1038/sj.bjc.6602787 PMC2361680

[18] Li CI. Risk of mortality by histologic type of breast cancer in the united States. Horm Cancer. 2010;1:156–65. 10.1007/s12672-010-0016-8. 21761358 10.1007/s12672-010-0016-8 PMC10357995

[19] Oladeru OT, Singh AK, Ma SJ. Association of endocrine therapy with overall survival in women with hormone receptor-positive, HER2-negative, node-negative breast cancer of favorable histology. Breast J. 2020;26:2006–10. 10.1111/tbj.13995. 32741050 10.1111/tbj.13995

[20] Sasada S, Kimura Y, Masumoto N, Emi A, Kadoya T, Arihiro K, et al. Breast cancer detection by dedicated breast positron emission tomography according to the world health organization classification of breast tumors. Eur J Surg Oncol. 2021;47:1588–92. 10.1016/j.ejso.2021.02.026. 33685728 10.1016/j.ejso.2021.02.026

[21] Bitencourt AGV, Graziano L, Osório CABT, Guatelli CS, Souza JA, Mendonça MHS, et al. MRI features of mucinous cancer of the breast: correlation with pathologic findings and other imaging methods. AJR Am J Roentgenol. 2016;206:238–46. 10.2214/AJR.15.14851. 26797349 10.2214/AJR.15.14851

[22] Fujii T, Yajima R, Kurozumi S, Higuchi T, Obayashi S, Tokiniwa H, et al. Clinical significance of 18F-FDG-PET in invasive lobular carcinoma. Anticancer Res. 2016;36:5481–5. 10.21873/anticanres.11129. 27798919 10.21873/anticanres.11129

[23] Gilardi L, Airò Farulla LSA, Curigliano G, Corso G, Leonardi MC, Ceci F. FDG and non-FDG radiopharmaceuticals for PET imaging in invasive lobular breast carcinoma. Biomedicines. 2023;11:1350. 10.3390/biomedicines11051350. 37239021 10.3390/biomedicines11051350 PMC10215865

[24] Groheux D, Giacchetti S, Moretti JL, Porcher R, Espié M, Lehmann-Che J, et al. Correlation of high 18F-FDG uptake to clinical, pathological and biological prognostic factors in breast cancer. Eur J Nucl Med Mol Imaging. 2011;38:426–35. 10.1007/s00259-010-1640-9. 21057787 10.1007/s00259-010-1640-9

[25] Bos R, van der Hoeven JJM, van der Wall E, van der Groep P, van Diest PJ, Comans EFI, et al. Biologic correlates of 18Fluorodeoxyglucose uptake in human breast cancer measured by positron emission tomography. J Clin Oncol. 2002;15:379–87. 10.1200/JCO.2002.20.2.379. 10.1200/JCO.2002.20.2.379 11786564

[26] Buck AK, Schirrmeister H, Mattfeldt T, Reske SN. Biological characterisation of breast cancer by means of PET. Eur J Nucl Med Mol Imaging. 2004;31(Suppl 1):S80–7. 10.1007/s00259-004-1529-6. 15127240 10.1007/s00259-004-1529-6

