# Supplementary material for: Prognostic value of FDG PET/CT in special types of breast cancer with non-favorable histology
Source: Discov Oncol. 2025 Jul 31;16:1450. doi: 10.1007/s12672-025-03278-5 (PMC12314144; doi:10.1007/s12672-025-03278-5)
Supplement: Supplementary file 1 — Supplementary Material 1 [file 12672_2025_3278_MOESM1_ESM.docx]

Table S1 T-test on the data in Reference 8 and present data

|  | | Kadoya [8] | | | Present data | | |  |  |  |
| --- | --- | --- | --- | --- | --- | --- | --- | --- | --- | --- |
|  |  | n | SUVamx(mean) | SD | n | SUVmax(mean) | SD | t value | df | *P*^1^ |
| T Stage | T1 | 241 | 2.76 | 2.00 | 54 | 2.54 | 1.67 | 0.855 | 90.7 | 0.395 |
|  | T2 | 101 | 5.83 | 4.12 | 48 | 5.03 | 4.68 | 1.02 | 82.8 | 0.311 |
|  | T3 | 2 | 4.86 | 1.31 | 11 | 4.62 | 3.52 | 0.172 | 4.56 | 0.871 |
|  | T4 | NA | NA | NA | 8 | 6.34 | 6.17 | NA | NA | NA |
| N Stage | N0 | 261 | 3.47 | 3.03 | 68 | 3.55 | 3.97 | -0.149 | 88.3 | 0.882 |
|  | N1 | 67 | 3.95 | 2.76 | 33 | 4.18 | 3.50 | -0.337 | 52.2 | 0.737 |
|  | N2 | 12 | 5.48 | 4.49 | 14 | 4.14 | 3.01 | 0.881 | 18.7 | 0.389 |
|  | N3 | 4 | 6.57 | 5.21 | 6 | 7.08 | 5.29 | -0.152 | 6.65 | 0.884 |
| Nuclear grade | NG1 | 79 | 2.94 | 1.99 | 27 | 2.31 | 2.47 | 1.20 | 38.2 | 0.238 |
|  | NG2 | 122 | 3.27 | 3.12 | 41 | 3.7 | 2.46 | -0.902 | 86.6 | 0.370 |
|  | NG3 | 142 | 4.43 | 3.46 | 52 | 5.06 | 4.92 | -0.850 | 70.3 | 0.398 |
| ER | Positive | 292 | 3.43 | 2.77 | 99 | 3.84 | 3.52 | -1.054 | 141.4 | 0.294 |
|  | Negative | 52 | 5.01 | 4.40 | 22 | 4.52 | 5.15 | 0.390 | 34.6 | 0.699 |
| PgR | Positive | 256 | 3.33 | 2.67 | 91 | 3.55 | 2.39 | -0.730 | 175.4 | 0.466 |
|  | Negative | 88 | 4.68 | 4.00 | 30 | 5.22 | 6.43 | -0.423 | 39.9 | 0.674 |
| HER2 | Positive | 47 | 5.01 | 3.56 | 13 | 3.88 | 2.32 | 1.367 | 29.5 | 0.182 |
|  | Negative | 297 | 3.46 | 2.99 | 108 | 3.97 | 4.00 | -1.208 | 152.6 | 0.229 |

^1^ T-test *P*-value, * significant *P*-value < 0.05

**Table S2** A chi-squared statistical test to analyse the distribution of molecular subtypes among histological subtypes

|  | | ILC | IMPC | Apocrine | Mucinous | Tublar | Metaplastic | Neuroendocrine | Adenomyo  -epithelial | Papillary ca. |
| --- | --- | --- | --- | --- | --- | --- | --- | --- | --- | --- |
|  | | n=39 | n=35 | n=15 | n=14 | n=6 | n=5 | n=3 | n=3 | n=1 |
| Subtype | HR+HER- | 3.00* | 2.17* | -5.29* | -1.01 | 0.47 | -0.80 | 1.01 | -1.70 | -1.75 |
|  | HER+ | -2.00* | 0.16 | 0.35 | 3.21* | 0.48 | -0.79 | -0.61 | -0.61 | -0.35 |
|  | HR-HER- | -1.95 | -2.84* | 6.27* | -1.61 | -1.02 | 1.71 | -0.71 | 2.66* | 2.48* |

* significant chi-sqiared value: |value|< 1.96
